# Supplementary figures and images for: The Cytotoxic Potential of Humanized γδ T Cells Against Human Cancer Cell Lines in In Vitro
Source: Cells. 2025 Aug 4;14(15):1197. doi: 10.3390/cells14151197 (PMC12345735; doi:10.3390/cells14151197)

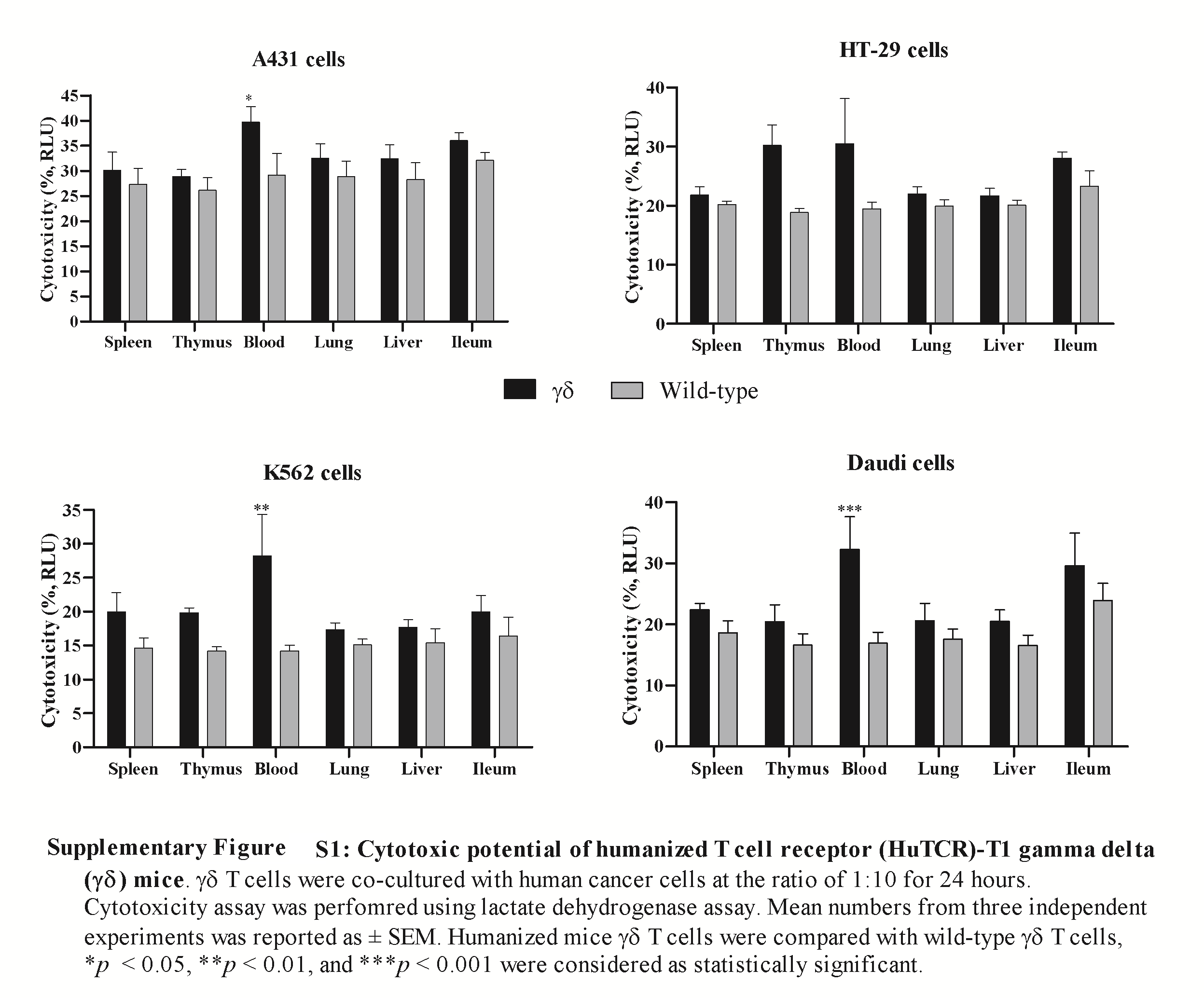

Supplement: Supplementary file 1 [file cells-14-01197-s001.zip › cells-3789864-supplementary.tif]
